# The role of spiritual mindset and gender in small business entrepreneurial success

**DOI:** 10.3389/fpsyg.2022.1082578

**Published:** 2022-12-23

**Authors:** Clara Margaça, Jose C. Sánchez-García, Giuseppina Maria Cardella, Brizeida R. Hernández-Sánchez

**Affiliations:** ^1^University of Salamanca, Salamanca, Spain; ^2^University of Coimbra, Coimbra, Portugal

**Keywords:** entrepreneur, small business, spirituality, pandemic (COVID-19), Portugal

## Abstract

**Introduction:**

Spirituality can be understood as a capital based on individual capabilities created by the application of intrinsic spiritual values, in order to use and develop human potential. The literature points out that spiritual capital increasingly influences and motivates entrepreneurs.

**Methods:**

In this paper, we investigate whether spirituality has a mediating role between psychological resilience, optimism and entrepreneurial success, and verify the gender differences. Our hypotheses are quantitatively tested on a sample of 233 micro and small Portuguese business owners during the pandemic crisis.

**Results:**

The main findings highlight that, while optimism and psychological resilience present a positive and significant relationship with entrepreneurial success in both genders, spirituality only impacts female entrepreneurial success.

**Discussion:**

Our study theoretically and empirically shows that the psychological resources and spirituality can be incorporated into new or existing programs designed to provide entrepreneurs with information on coping skills and how to engage in positive reorientation and reappraisal. In so doing, it improves the knowledge of the importance of psychological resources for the micro and small business’ recoverability during the pandemic, which is deeply rooted in the entrepreneurial ability to excel during adversity.

## 1. Introduction

Enterprises are the fundamental building blocks of society and the economy, and the largest group is made up of micro and small businesses. In the same way, analyzing the process of developing entrepreneurial success is also fundamental, because it is a driver of the necessary change for economic development and innovation (Al Issa, 2021). According to PORDATA (2019), in Portugal there was 44,492 small enterprises and 1.281.857 micro enterprises. Micro-businesses are of tremendous importance in almost all sectors and are an integral part of our business fabric. The micro and small enterprises sector becomes a key factor in the development of the local, regional and national economy (Audretsch and Link, 2012). This fact is due to its strategic role in promoting employment, innovative business, which, consequently, stimulates the increase in gross domestic product (Hangraeni et al., 2019).

The COVID-19 pandemic has had significant effects on economies (Fernandes, 2020), and, almost three later, is difficult to estimate the economic impacts (Zhang et al., 2020). The pandemic restrictions were more severe on micro and small enterprises (Korankye, 2020; Shafi et al., 2020). For instance, this type of business, according to Liu and Cheng (2018), have lower capital reserves, less inventory, and lower productivity, rendering them more vulnerable to crises. Considering the changes in business processes caused by the pandemic, micro and small companies may prove to be less resilient and more vulnerable when dealing with the various associated costs and barriers (Korankye, 2020). The transition to teleworking can be seen as an obstacle, given the low level of digitalization and the complexity of integrating the technology of these businesses. A study carried out, by this author, with micro and small companies in Ghana revealed that the tourism sector, the hotel industry and restaurants were the most affected by the pandemic, as they showed a significant decline in their benefits. At the business level, there is no doubt that the sector that suffered the greatest impact was that of micro and small companies. According to Cowling et al. (2010), we can assess them in terms of social, economic and psychological impacts. However, most studies on entrepreneurship and the pandemic have only focused on the economic effects.

Gender is a contextual and influential factor in the sphere of values and actions and, according to Risman, can be considered a social structure, which “indirectly shapes actors’ perceptions of their interests and directly by restricting choice” (Risman, 2004, p. 432), with individual, organizational and institutional impact (Borquist and de Bruin, 2019). A study by Lewis (2017) highlighted that, for women, business is an extension of themselves and a way to improve their self-esteem and self-concept, as well a way to making meaning. According Koltai et al. (2020), in 2019, more than 10 million (33%) women were entrepreneurs in the European Union Member States; Portugal appears in second place in the statistics with 39% of women entrepreneurs. In the last quarter of 2022, the European Medicines Agency continues to warn that COVID-19 it is not yet over, and this type of crisis can trigger a shortage of family resources, as well as shape their attitudes, changing the way they deal with the economic consequences compared to men. For instance, according UN WOMEN (2020), the pandemic has had a significant impact on microenterprises of females, due to were closed for a period at the beginning of the crisis. On the one hand, these differences are seen as gender stereotypes, that is, the business world is still seen as belonging to the males, which increases the favorability of their models of behavior (Feder and Nițu-Antonie, 2017). And on the other hand, according Brush (2006), these differences can be seen as positive, in the sense of a broader contribution and in different business sectors, as well as for the development of society.

With de aim to fill this gap and in accordance with recent increase in scholarly attention devoted to exploration of the role of psychological resources in entrepreneurship (Baluku et al., 2018), our study supplies unique insight into psychological resources as predictors of entrepreneurial success. Moreover, Psychology conceptualizes crisis as a life event that an individual perceives as stressful to the extent that normal coping mechanisms are insufficient. More than that, this science explores the several mechanisms of facing a crisis, such as traumatic losses, catastrophes that the pandemic itself can cause (Dobrodolac et al., 2018). This article contributes to this domain by highlighting the value of psychological strengths to business success and reflect on gender differences.

Using a survey data from 233 micro and small Portuguese business owners, we examine the relationship between the optimism and psychological resilience and entrepreneurial success as well as de mediating effect spirituality on this relationship. The main findings show a positive and significant effect of optimism on entrepreneurial success, and an association between spirituality and entrepreneurial success in both genders. In addition, spirituality was presented as a positive mediator between psychological resilience and success and between optimism and success, but only in females. Hence, we indicate that is crucial to pay attention to the individual and their idiosyncrasies, because often wealth is not their primary motive for achieving entrepreneurial success (Chu, 2007; Rindova et al., 2009), but entrepreneurs may also utilize additional sources of inner guidance (e.g., spirituality), creating both tangible and intangible value.

Our study provides several contributions to the debate on micro and small business recuperation during the pandemic by highlighting how entrepreneurs face adversity and the measures imposed. We suggest how the entrepreneurs’ psychological resources may generate an alternative and accurate way to achieve success, i.e., the inclusion of spiritual factors, for instance, in entrepreneurial scholarship allows for investigation into the full dimensionality of success, contrary to purely economic research work (Kauanui et al., 2010). In so doing, we also make one of the first efforts to empirically identify and measure different types of psychological resources (Bockorny and Youssef-Morgan, 2019; Chadwik and Laver, 2020). Furthermore, this article adds to ongoing scholarly debate on the role of economic versus individual gains in achieving entrepreneurial success during a pandemic time. Undoubtedly, if we think that an individual’s intrinsic spirituality allows them to find passion in their work, and persevere in their entrepreneurial activity, our study suggests that entrepreneurs can benefit from this connection to overcome adversity, regardless of gender.

## 2. Theoretical background

### 2.1. Psychological resources: A path to entrepreneurial success

Successful entrepreneurs are commonly characterized as those who have mastered the art of learning to learn, and put it into practice, in dynamic environments of change and uncertainty (Young, 2007). Entrepreneurs of micro and small companies face the success of their activity differently when compared to entrepreneurs of large companies. This may be due to the fact that micro and small entrepreneurs establish a bond, a psychological involvement with their company. In this way, psychological resources can be seen as explanatory factors of entrepreneurial success, in particular, in micro and small companies (Gorgievski et al., 2011). There is a consensus (e.g., Wach et al., 2018) that entrepreneurial success is not just financial, but can include, for example, personal fulfilment. An optimistic entrepreneur creatively and more accurately explores business opportunities and believes they have a greater ability to control (Krueger, 2007). Psychological resources can be seen as precursors or maintainers of entrepreneurship. Hence, individuals who have higher psychological resources tend to perform higher as they use these resources to overcome obstacles (Hobfoll, 2002). For instance, the successive economic crises that countries are going through lead the individual to recognize that the ‘profit’ factor is not the main motivator/precursor for de decision-making to become an entrepreneur (Katz, 1992; Amit et al., 2001). According to Baluku et al. (2018), to some extent, business success results from psychological resources or states that entrepreneurs invest in their work.

Optimism and resilience together as individual psychological skills can produce higher levels of coping, as allow the individual a “positive assessment of circumstances and probability of success based on motivated effort and perseverance” (Luthans et al., 2007a, p. 550). According to Hmieleski and Carr (2008), this use of optimism and psychological resilience is positively related to entrepreneurial performance and, in addition, supports the efforts applied in entrepreneurship (Baron et al., 2016).

As important as studying entrepreneurial success according to an economic parameter is the study of subjective success, that is, the focus on associated psychological processes and resources (Baluku et al., 2018). Hence, according to Patel and Thatcher (2014), psychological attributes can be a key resource to preserve and achieve entrepreneurial success. There is no doubt that optimism increases motivation and direction towards a certain goal, and psychological resilience allows individuals to plan alternative pathways to achieve goals and adapt to the adversities of the entrepreneurial process. Being spiritual does not imply practicing a religion, however, individuals can equally have a strong spiritual value system, an “inner experience to connect with a higher power” (Amin Mohamed et al., 2004, 106). Spirituality involves a high level of cognitive processes (Mubarak et al., 2014), and integrates the entrepreneur’s moral, social and religious values in driving the success of his business (Kolsome, 2010), especially in women (Borquist and de Bruin, 2019). For instance, spiritual mindset is an important guide and strategy of entrepreneurial orientation and leadership among women (Borquist and de Bruin, 2019; Latukismo et al., 2021).

### 2.2. The impact of optimism and psychological resilience In entrepreneurs

According to Waters et al. (2021), positive psychology factors proved to have a leading role in personal strengthening through adversity, namely intrapersonal variables such as optimism (Prati and Pietrantoni, 2009). The perspective of positive psychology focuses on the real and potential capabilities of individuals, which allows us to understand how they deal with adversity and grow in times of crisis (Waters et al., 2021). Optimism is understood as a prerequisite during hard times (Al Issa, 2021), and can stimulate a desired behavior in order to promote entrepreneurial success, aiding in risk management. Optimism relates to a flexible set of adaptive strategies, and is considered a predictor of an individual’s ability to manage and cope with the adversities of a potential traumatic event (Benight and Bandura, 2004), like a pandemic. Zoellner and Marcker (2006) suggest that accepting situations considered unchanged and reassessing a crisis event in a positive light allows for personal growth. Previous works (e.g., Holland and Shepherd 2013; Cardon and Kirk 2015) reinforce the idea that optimism is one of the factors that most encourage entrepreneurs. According to Storey (2011) “key empirical regularities among new and small firms are explained more insightfully by elevating the role of chance and combining it with the optimism of the business owner” (p. 317), driving the persistence to persevere in a business (Brown and Marshall, 2001).

Psychological resilience is, the flexible capacity to bounce back from negative experiences and adapt to the changing demands of stressful scenarios (Lazarus, 1993). According to Davidsson and Gordon (2016), entrepreneurs will be more successful if they are resilient. For instance, a cross-sectional study carried out with workers in Canada found that resilience is one of the factors that is positively related to prosperity at work (Pacheco et al., 2020). Resilient individuals are better able to remain optimistic (Mak et al., 2011), due to positive emotions are the foundation of psychological resilience. At the cognitive level, resilient people better assess threats (Fletcher and Sarkar, 2013), and tend to engage in activities with a future perspective (Masten, 2001). Entrepreneurs can profit from this resource, as the ability to interpret (positive) and respond to the stressful event can dictate the success of their venture (Duchek, 2017).

*H1:* For both genders – Psychological resilience has a significant and positive effect on entrepreneurial success.

*H2:* For both genders – Optimism has a significant and positive effect on entrepreneurial success.

### 2.3. The mediating role of intrinsic spirituality

Several health studies show that there is a relationship between this and religious indices, such as attendance at worship places, and a self-assessment of religiosity and spirituality (Pargament, 1997; Koenig et al., 2001). Starting from the premise that people are able to cope with traumatic events (e.g., illness) through religion or spirituality, it is remarkable and imperative to test its effect also on growth after a moment of crisis, such as the pandemic, in relation to its entrepreneurial career. The study conducted by Prati and Pietrantoni (2009) revealed that spirituality moderately predicted positive changes after a crisis event, concluding that it provides a sense of community (Pargament et al., 2004), and personal beliefs drive the process of meaning/fulfilment and coping (Cadell et al., 2003).

Spirituality presupposes a connection to a superior force capable of helping the person to overcome daily difficulties and negative circumstances. It is an experiential process whose characteristics include relation with nature or with one or more spiritual forces, in the search for meaning and the purpose of ‘things’ (social phenomena and issues, in the Durkheimian sense). Inserted in a specific sociocultural context - which implies behaviours, experiences, interpersonal relationships and the search for values -, it may or may not include participation or formal religious activity, that is, an institutionalized relation with a certain religion, religious movement or religious denomination (church). It has been attributed a greater importance to the individual’s spirituality, especially with regard to resilient strategies in the face of adverse situations, in the way it interferes in the level of disease/health, and in learning (Rodrigues, 2007), but also, at the present time, in more and better capacity to initiate and develop an entrepreneurial activity. For instance, several studies point to another facet of spirituality, as a coping strategy to face stressful business situations (e.g., Herriott et al., 2009). Or also as an intrinsic drive and motivation for people to find meaning in their work (Giacalone and Jurkiewicz, 2003).

If we think of spirituality as a capital (like human or social capital), it can be defined, according to Liu (2015), as the power and influence arising from an individual spiritual belief and practice. That is, spiritual capital is based on individual capacities created by the application of intrinsic spiritual values. Based on the idea that spiritual capital is the search for meaning with a view to the development of human potential, it is based on this capacity that an individual chooses their personal and professional guidelines (Zohar, 2010). According to Middlebrooks and Noghiu (2010) this practice emphasizes the post-capitalist economy, based on values over capitalist culture/profit maximization. Currently, it is clear that this philosophy increasingly influences and motivates entrepreneurs.

In Silicon Valley, where world-renowned start-ups are located, there is a church called Vive, which offers tranquillity in a hyperconnected world, arguing that the “start-up of Jesus was the manger.” Entrepreneurs at companies such as Tesla, Lyft, “settle the score” between technology and their relationship with it, reconnecting with the full life and preparing to be leaders of extraordinary lives. Recently, the literature has pointed out (e.g., Lari, 2012; Mubarak et al., 2014) that spirituality affects entrepreneurial motivation and perseverance in this process, what will dictate the success of the enterprises. This internal dimension can increase well-being and quality of life, as well as commitment and productivity (Karakas, 2010) and, consequently, entrepreneurial success (Pio, 2010). In addition, Zsolnai (2019) proposes the idea of a “spiritually informed economy,” which aims to integrate both material and immaterial aspects, that is, the different goals and objectives, including the spiritual ones.

*H3:* For both genders – Spirituality mediates the positive effect between psychological resilience and entrepreneurial success.

*H4:* For both genders – Spirituality mediates the positive effect between optimism and entrepreneurial success.

## 3. Materials and methods

### 3.1. Sample and data collection

To explore the changes during the COVID-19 pandemic, we collected data generated by a survey of the entrepreneurs of micro and small Portuguese firms. We followed the European definition of micro enterprise (less than 10 workers) and small enterprise (less than 50 workers). Among the 28 countries of the union, the vast majority (93%) of SMEs are micro, while another 5.9% are small companies. Portugal is in second place with the aforementioned 99.3% (more specifically around 1.2 million micro-enterprises). These values correspond to around 30% of GDP and 40% of employment.

The data collection process started with an email invitation to participate in an online survey. The target was the entrepreneur (the owner) because of their desirable capacity to provide data on firm-related as well as personal-oriented questions. We received 233 responses from a universe of 800 emails sent (response rate 12.44%). Regarding nationality, the random sample is composed of a majority of Portuguese entrepreneurs, 5.6% Brazilians and 0.4% Colombians, with age varying between 24 and 73, and average age of 43 years (SD = 11.01). We can consider that this is an equitable sample in terms of gender, with 47.2% males and 52.8% females, and more than half (64.5%) have higher education. With regard to the business sector, 14.2% are dedicated to restaurant and hotels, 13.7% to health and wellness services, 10.7% are dedicated to food retail, 10.3% to services, 9.4% to the tourism sector and 9.0% to craftsmanship. The remainder concerns areas such as energy and sustainability, commerce, technology and information, fashion/textile, agriculture, among others. 52.4% of respondents attested to having opened their business for more than 5 years, and 9.4% for less than a year - which means that these businesses were opened during the pandemic. Most companies are located in the central region of Portugal (41.6%) and in the Lisbon metropolitan area (28.3%). When we focus on the spirituality dimension, 60.1% of the respondents consider themselves a spiritual person, and, although 54.5% do not follow a religious system, 65.7% believe that spirituality is a strategy to cope a crisis, like the one we are currently experiencing. The detailed description of the sample is reported in Table 1.

**Table 1:** Sample description [*N* = 233].

	Frequency	%
**Gender**		
Male	110	47.2
Female	123	52.8
**Marital Status**		
Single	48	20.6
Married	111	47.6
*De facto* union	42	18.0
In a relationship	11	4.7
Divorced	21	9.0
**Education**		
Elementary School	5	2.1
Intermediate School	6	2.6
Middle School	6	2.6
High School	51	21.9
Professional Education	15	6.4
Degree	93	39.9
Master	40	17.2
PhD	9	3.9
MBA	2	0.9
Postgraduate studies	6	2.6
**Region**		
North	36	15.5
Centre	97	41.6
Lisbon Metropolitan Area	66	28.3
Alentejo	7	3.0
Algarve	5	2.1
Autonomous Region of the Azores	16	6.9
Autonomous Region of Madeira	6	2.6
**BeliefSystem_follower**		
Yes	106	45.5
No	127	54.5
**Spiritual Person**		
Yes	140	60.1
No	93	39.9
**Spirituality_Crisis**		
Yes	153	65.7
No	80	34.3
**Business Sector**		
Services	24	10.3
Health and wellness	32	13.7
Restaurant and Hotels	33	14.2
Food retail	25	10.7
Craftsmanship	21	9.0
Tourism	22	9.4
Energy and Sustainability	17	7.3
Technology and Information	12	5.2
Others	47	20.2
**Business_opening**		
Less than 1 year	22	9.4
Between 1 and 3 years	42	18.0
Between 3 and 5 years	47	20.2
More than 5 years	122	52.4

### 3.2. Measure of constructs

*Subjective Entrepreneurial* Success (SES, *α* = 0.79) was measured through the homonymous scale developed by Dej et al. (2009). This scale is composed by 24-item (Likert-point scale 1 – strongly disagree and 5 – strongly agree). Using this scale, it is possible to evaluate: (i) the importance of entrepreneurial success criteria; (ii) their level of achievement; and (iii) the mismatch between importance and achievement of success criteria. Subjective entrepreneurial success can be understood as the assessment that entrepreneurs make regarding how they perform their activities, considering personal values, in relation to their own goals (Gorgievski et al., 2011).

*Psychological Resilience* (PsyResil, *α* = 0.81) and *Optimism* (Opt, *α* = 0.91) scales were extracted from the Entrepreneurial Orientation Questionnaire (Sánchez-García, 2010). Psychological Resilience (9 items) can be considered as an ability to cope with adversities and recovering from adverse experiences, a set of continuous behaviors, formed by the fusion of the following personal behavioral characteristics: flexibility, high motivation, perseverance, and optimism. This fact gives an entrepreneur with discernment the ability to adopt the application of different strategies to deal with a challenge until it is overcome (Margaça et al., 2020). We measure PsyResil by asking entrepreneurs, for instance, “I think I can grow positively when facing difficult situations.” Regarding Optimism (10 items), this variable frames the level of agreement in which a person believes that their future holds positive outcomes, or that there is a positive side of every experience. An item example is: “No matter how bad things can go, I always find something positive.”

*Intrinsic Spirituality* (ISpirit, *α* = 0.95): We used the modified six-item Intrinsic Spirituality Scale (Hodge, 2003) that measures the degree to which spirituality functions as an individual’s master motive, for theistic and non-theistic populations, both within and outside of religious frameworks. The scale uses a sentence completion format to measure various attributes associated with spirituality. That is, an incomplete sentence fragment is provided, followed directly below by two phrases that are linked to a scale ranging from 0 to 10. The range provides with a continuum on which to reply, with 0 corresponding to absence or zero amount of the attribute, while 10 corresponds to the maximum amount of the attribute (e.g., In terms of the questions I have about life, my spirituality answers; 0 – no questions and 10 – absolutely all my questions). The sentence completion format measures various attributes associated with spirituality; that is, an incomplete sentence fragment is provided, followed directly below by two phrases that are linked to a scale ranging from 0 to 10. The range provides with a continuum on which to reply, with 0 corresponding to absence or zero amount of the attribute, while 10 corresponds to the maximum amount of the attribute (Hodge, 2003).

*Control variables:* To control for other factors that may influence main hypothesized relationships, we used several control variables drawn from the extant literature. Specifically, we controlled for if the person considers spirituality as a resource to cope with crisis (dichotomous variable), business sector, and age of the business. Spirituality has been viewed, in several areas, as a coping resource and entrepreneurship is beginning to be an exception. Several studies have highlighted the position and key role of spirituality in business success (e.g., Grine et al., 2015). Experience in a particular business sector increases the chances of success in obtaining profits and in the survival of the company (Van Praag, 1997). In the same way, Bilan et al. (2020) find that the duration of business activities increases the chances of entrepreneurial success.

## 4. Analysis and findings

To analyze the proposed model, Structural Equation Modelling was used. We used IBM SPSS Amos 26 and IBM SPSS 26 for the remaining analyses. The following indices are used: the Comparative Fit Index (CFI > 0.90), the Adjusted Goodness of Fit (GFI > 0.95; Hair et al., 2010); the Root Square Error of Approximation (RMSEA <0.05), the Tucker–Lewis Index (TLI > 0.90; Awang, 2012), and the Expected Cross Validation Index (ECVI: the lower the index, the better the fit and the better the model can predict the future covariance of the sample (Browne and Cudeck, 1992)), due to, according to Kline (2011), the sample is greater than 200. Lastly, multiple squared correlations (*R*^2^) were made to demonstrate how much of the variation in the independent variables is explained by the predictors.

Model fit indices for the proposed model resulted in: CFI = 0.994; TLI = 0.923; GFI = 0.975; RSME = 0.031; ECVI = 0.444. These results reveal a good fit and above the common standards (Browne and Cudeck, 1992; Hair et al., 2010; Awang, 2012). Regarding the variance of the dependent variable, the R^2^ explains in the group of females 62% and in the group of males 53%. In this way, the results achieved allow us to recognize the necessary theoretical coherence. We show in Table 2 the correlations, which reveal that the model and hypothesis interactions maintain the analysis criteria.

**Table 2:** Correlations.

	1	2	3	4	5	6	7
1. PsyResil	1.00						
2. Opt	0.396**	1.00					
3. ISpirit	0.266**	0.366*	1.00				
4. SES	0.479**	0.536**	0.472**	1.00			
5. Business Sector	0.168*	0.108*	0.022	0.008	1.00		
6. Business opening	0.093	0.055	0.049	0.057	0.106	1.00	
7. Spirit_Crisis	0.163*	0.231	0.558**	0.109	0.008	0.135*	1.00

**p < 0.01; *p < 0.05.

We used Maximum Likelihood Estimate, in order to estimate the coefficient and significance of direct effects. To analyze mediation effects and group differences, Bootstrap was used with 2000 iterations and 0.95 bias-correction. In the Table 3 it is possible to show each regression to understand how each one interacts individually, including control variables. In one hand, the Optimism, for instance, has a stronger regression value, for both gender females and males. Intrinsic Spirituality, on the other hand, has a strong value only for females. Psychological Resilience effect on entrepreneurial success is drastically stronger and significant on males. Regarding our control variables, business opening effect is positive and significant on Entrepreneurial Success in females, and the business sector in males. Spirit_Crisis presents a significant effect on entrepreneurial success for both males and females.

**Table 3:** Regression weights.

	Females			Males		
	*B*	SE	*p*	*B*	SE	*p*
ISpirit ← Opt	0.191	0.036	***	0.058	0.068	0.598
ISpirit ← PsyResil	0.152	0.043	***	0.214	0.064	0.189
ISpirit ← Spirit_Crisis	0.162	0.041	***	0.374	0.069	***
ISpirit ← Bus_Sector	−0.255	0.464	0.339	0.054	0.073	0.785
ISpirit ← Bus_Open	0.143	0.039	0.021*	0.169	0.056	0.031*
SES ← Opt	0.533	0.057	***	0.653	0.066	***
SES ← PsyResil	0.132	0.041	0.003*	0.433	0.079	***
SES ← Spirit	0.298	0.044	***	0.148	0.072	0.112
SES ← Spirit_Crisis	0.078	0.052	0.0029*	0.151	0.221	0.019*
SES ← Bus_Sector	−0.192	0.543	0.633	0.089	0.386	0.031*
SES ← Bus_Open	0.113	0.039	0.035*	0.384	0.065	0.237

**p < 0.001 or less; *p < 0.05.

Table 4 presents the results obtained from our path model by females and males. Intrinsic Spirituality mediates an effect between Psychological Resilience and Entrepreneurial Success in both genders, but a very positive and significant in females. Although less strong, the relationship between Optimism and Entrepreneurial Success is also mediated by Spirituality only in females. Lastly, Table 5 shows the mean for each variable by gender, as well results obtained from the t-test analysis for differences. To compare the mean difference between both genders, the t-Test statistic was used. Accordingly, Levene’s test was used to observe whether there was homogeneity within each variable. Variables that yielded statistically significant results (<0.05), were analyzed under the assumption that are not homogeneous. The biggest difference in response comes from Spirituality, with a mean difference of 0.553 (significant, *p* < 0.001), and the smallest from Entrepreneurial Success (significant, *p* < 0.001).

**Table 4:** Effects for path model by gender.

	Females				Males			
	Effects		CI		Effects		CI	
	*B*	*p*	Lower	Upper	B	*p*	Lower	Upper
Opt → SES	0.398	***	–	–	0.583	***	–	–
PsyResil → SES	0.121	0.003*	–	–	0.311	0.007**	–	–
ISpirit → SES	0.183	***	–	–	0.142	0.044*	–	–
psyResil → ISpirit → SES	0.124	***	0.013	0.064	0.032	0.047*	0.000	0.081
Opt → ISpirit → SES	0.024	0.029*	0.006	0.062	0.014	0.421	−0.016	0.059

**p < 0.001 or less; **p < 0.01; *p < 0.05.

**Table 5:** Mean difference by gender.

	Gender	Mean by gender			*t*-test for equality		
		Mean	SD	SE	*t*	*p*	Mean dif.
PsyResil	Female	3.879	0.697	0.022	−2.293	0.017	−0.198
	Male	3.681	0.763	0.038			
Opt	Female	3.554	0.489	0.017	0.521	0.001	0.248
	Male	3.802	0.453	0.014			
ISpirit	Female	5.342	3.045	0.109	−3.243	0.001	0.553
	Male	4.789	2.867	0.112			
SES	Female	3.568	0.367	0.012	2.386	0.001	0.021
	Male	3.547	0.498	0.019			

## 5. Discussion and conclusion

Recently, several studies have been carried out on how small businesses react to crisis and how resilience works (e.g., Williams et al., 2017; Doern et al., 2019), which reveal that management and adaptation capacity is extremely important for the company survival. However, the pandemic caused by COVID-19 came suddenly and companies had to try to quickly adapt to the lockdown and the delayed support measures. Our study proposes that a plausible explanation for entrepreneurial success may lie in the psychological resources during the pandemic and not just economic gains. More specifically, in the idea that spirituality can be a mediator of resilience and optimism to overcome the implications caused by crisis scenarios. Hence, following the lead of recent researchers who argue that psychological resources are positively related to entrepreneurial success (Williams et al., 2013; Baluku et al., 2018), in addition to pointing out the need to dig into spirituality as a predictor of positive changes after a crisis (e.g., Prati and Pietrantoni, 2009) and that individual beliefs lead to success and achievement, in addition to acquiring the form of a coping strategy, we investigate how optimism, psychological resilience and spirituality can predict the entrepreneurial success of small business owners and we verified the gender differences.

First, our main findings suggest that the personal attributes of small entrepreneurs, such as optimism and psychological resilience, directly and indirectly influence the success of their company, especially during the restrictions related to the pandemic that we are still facing. As such, results of this study offer new insights into the entrepreneurs’ success and suggests that it is significantly influenced by positive psychological related factors. More specifically, our findings show the existence of a positive and significant relationship between optimism and entrepreneurial success (both in males and females). A possible explanation for this lies in the idea that there is a decrease in gender differences in entrepreneurial self-perceptions with growing involvement in entrepreneurial activities (Malach-Pines and Schwartz, 2007), and, consequently, personal characteristics help in this process. Furthermore, optimistic entrepreneurs capitalize on opportunities, because they believe that their chances of success are greater than others (Shane and Venkataraman, 2000). More precisely, by considering optimism and resilience as resources of coping (Luthans et al., 2007a), results corroborate our initial view that positively evaluating a situation greatly increases the likelihood that entrepreneurs will preserve during a crisis situation. Based on the premise that psychological resilience captures the organization’s ability to maintain “reliable” functioning during a crisis (Williams et al., 2017), this resource allows for creatively responding to adversity, and developing alternative ways of doing business and recover (Luthans et al., 2010; Linnenluecke, 2017). Specifically, the findings show a positive and significant effect of psychological resilience on entrepreneurial success for both males and females. However, we owe special attention to masculine values. We believe that this may be due to the fact that there is the conflict between work and family domains plays an important role in the perceived entrepreneurial success among males and females (De Simone et al., 2021). Alternatively, women seem to place importance on other resources to achieve a balance between entrepreneurial success and family life. Our analyses show that spirituality impacts significantly on entrepreneurial success in females, but not in males. Despite efforts, the trend is still that men are more involved in entrepreneurial activities than women and they face more challenges when compared to men (Mehtap et al., 2019). However, this could offer an alternative theoretical explanation to account for why some internal-related aspects represent an asset for business issues.

Second, we explore whether the mediating effect of spirituality acts on entrepreneurial success when we refer to resilient and optimistic individuals. While we do not find statistical support for the moderating effect of the spirituality between optimism e entrepreneurial success for both genders (only for females), we find interesting insights regarding psychological resilience. In general, women entrepreneurs revealed that spirituality has a strong and positive mediating effect between psychological resilience and the success of their entrepreneurial activity. Entrepreneurial resilience is understood as the dynamic ability to resist and quickly overcome maladjustment and one of the important personal traits in entrepreneurship, helping in the progress of entrepreneurial activity (Bernard and Barbosa, 2016). Our study suggests that this recoverability during the pandemic requires resilience, which is deeply rooted in the entrepreneurial ability to excel during adversity. Likewise, optimism is seen as a source of faith that leads a person to believe in their entrepreneurial success capacity (Al Issa, 2021).

### 5.1. Theoretical contributions

This study contributes to the recent stream of studies on expanding the scope of entrepreneurial success (Gorgievski et al., 2011; Duchek, 2017; Bilan et al., 2020; Al Issa, 2021). Our theoretical contribution lies in the investigation of psychological outcomes including optimism, psychological resilience and spirituality, which allow us to highlight that they are a key determinant to entrepreneurial success of micro and small businesses, and have demonstrated that entrepreneurial success includes measurement of psychological resources (Hmieleski and Carr, 2008; Peterson et al., 2011; Duchek, 2017). In this way, this study also contributes to a deep understanding through which spiritual values can play a leading role when dealing with a context of crisis among micro and small business owners. Our findings demonstrate that psychological resources significantly contribute to the success of micro and small companies. More precisely, considering that entrepreneurial success is dynamic, it is important to assess this fluctuation of psychological states, which can determine and direct behavior in a scenario of uncertainty and challenges (Juhdi and Hamid, 2015). Our study also advances the literature on the gender differences in this context (De Simone et al., 2021). Hence, the contribution offered to the literature concerns both the understanding of what psychological resources used for both male and female to achieve entrepreneurial success while dealing with the pandemic.

### 5.2. Practical implications

Our findings offer several valuable insights into micro and small entrepreneurs. First, we believe our work has interesting implications also for researchers, entrepreneurs and policymakers, whom should adopt a more global perspective on entrepreneurial success. Second, the findings suggest that psychological resources and spirituality can be integrative and incorporated into new or existing programs designed to provide entrepreneurs with information on coping skills and how to engage in positive reorientation and reappraisal. Furthermore, the insights of our study point to the importance of focusing on the non-economic side of entrepreneurial success and offer a relevant contribution to business training, mentoring and counselling. In particular, entrepreneurs must be supported in developing their psychological resources. It is important that these important actors in society know how to apply them in the processes adjacent to their entrepreneurial activity, allowing them to develop their psychological capital (Luthans et al., 2007b) and, consequently, to entrepreneurial success. Finally, by illustrating the differential effects of spirituality, psychological resilience and optimism in the entrepreneurial context, we caution against adopting a purely economic perspective of business success to the detriment of an idiosyncratic view of the entrepreneur.

## 6. Limitations and future research

This study presents certain limitations that could be overcome in future research. Future studies could further explore whether – and to what extent - entrepreneurial success is related to the Self-Determination Theory (Deci and Ryan, 2000; Bilal et al., 2021). Moreover, we only use two separate variables of psychological capital as resources for success. Future studies could adopt a more nuanced approach, by using the introspective psychological inventory – Psychological Capital Questionnaire (PCQ; Luthans et al., 2007a). Psychological capital is, in a way, an expansion of the concept of “economic capital,” but it differs from human capital or social capital (Luthans et al., 2004). Psychological capital components are valuable and determinant personal resources for small business success (Runyan et al., 2007; Al Issa, 2021). Finally, in common with previous studies on entrepreneurial success which rely on cross-sectional data (e.g., Mubarak et al., 2014; Juhdi and Hamid, 2015; Duchek, 2017), we find that the nature of cross-sectional data makes it difficult to detect whether success that lasts over time. Hence, it would be also interesting for subsequent studies to further explore whether psychological resources could significantly influence micro and small entrepreneurs’ entrepreneurial success, using of a longitudinal approach or qualitative techniques.

## Data availability statement

The raw data supporting the conclusions of this article will be made available by the authors, without undue reservation.

## Author contributions

CM: writing, sampling, statistics, and discussion. JS-G: writing and discussion. GC and BH-S: discussion. All authors: contributed to the article and approved the submitted version.

## Conflict of interest

The authors declare that the research was conducted in the absence of any commercial or financial relationships that could be construed as a potential conflict of interest.

## Publisher’s note

All claims expressed in this article are solely those of the authors and do not necessarily represent those of their affiliated organizations, or those of the publisher, the editors and the reviewers. Any product that may be evaluated in this article, or claim that may be made by its manufacturer, is not guaranteed or endorsed by the publisher.
